# Why Our Youth Vape?—A Trend Analysis Based on Cross-Sectional Annual Surveys of Middle and High School Students in the U.S.

**DOI:** 10.3390/medicina62010223

**Published:** 2026-01-21

**Authors:** Tianyuan Guan, Zhaochong Yu, Preethi Bhosle, Chen Li, Kai Sun, Marepalli B. Rao

**Affiliations:** 1 College of Public Health, Kent State University, Kent, OH 44242, USA; 2 Department of Biostatistics, Health Informatics & Data Sciences, College of Medicine, University of Cincinnati, Cincinnati, OH 45221, USA; yuzc@mail.uc.edu (Z.Y.);; 3 Department of Exercise Science and Exercise Physiology, Kent State University, Kent, OH 44242, USA; cli42@kent.edu; 4 Department of Information Systems, University of Maryland, Baltimore County, Baltimore, MD 21250, USA; ksun1@umbc.edu

**Keywords:** youth vaping, e-cigarettes, tobacco trends, National Youth Tobacco Survey (NYTS), public health, adolescent behavioral health, nicotine use

## Abstract

*Background and Objectives*: E-cigarette use among middle and high school students steadily increased in the last decade, raising significant public health concerns. It is argued that e-cigarettes contain a lower level of toxicants than combustible tobacco cigarettes. This perception has contributed to the growing popularity of e-cigarettes among youth. However, lower level of toxicants does not mean addiction is less likely. In this study, we examine trends in the usage of electronic cigarettes among middle and high school students in the U.S. from 2018 to 2023, compare these patterns with traditional cigarette use over the same period and population, and explore the underlying reasons driving e-cigarette use within this demographic. Understanding patterns and motivations are essential for developing effective prevention and intervention strategies. *Materials and Methods*: Data were derived from the National Youth Tobacco Survey (NYTS), administered by the Centers for Disease Control and Prevention (CDC). Population-level estimates and standard errors were calculated to facilitate comparisons across subgroups and time points. *Results*: A declining trend in e-cigarette use was observed over the study period. The proportion of students who reported exclusive e-cigarette use was significantly higher than that of those who used combustible cigarettes exclusively. Among reported motivations, anxiety consistently ranked among the top three reasons for vaping for current users. *Conclusions*: The downward trend in youth e-cigarette experimentation is encouraging. However, the period from 2020 to 2023, which includes the COVID-19 pandemic (2020 and 2021) era, may have impacted usage patterns. Alarmingly, the number of students who vape exclusively is approximately five times greater than that of those who smoke only cigarettes. This finding contradicts claims by e-cigarette manufacturers that their products facilitate smoking cessation. Further rising e-cigarette smoking exclusively is of public health concern, which warrants targeted intervention.

## 1. Introduction

Electronic cigarettes, also referred to as vaping are a form of electronic nicotine delivery systems (ENDSs). They are one of the most popular means of substance use. Initially marketed as tools for smoking cessation, e-cigarettes have rapidly gained popularity, particularly among adolescents for recreational use [1,2]. E-cigarettes have been the most used tobacco product among U.S. middle and high school students since 2014, surpassing traditional cigarette smoking [3,4]. From 2011 to 2018, electronic cigarette use increased significantly among middle school (0.6% to 4.9%) and high school (1.5% to 20.8%) students [5]. By 2019, those numbers rose dramatically to 27.5% for high school students and 10.5% for middle school students [6]. Data collection on tobacco usage among middle and high school students in the U.S. was carried out from January to May every year in person until 2019. However, the study was conducted using an online survey during pandemic years and later. The selection of samples was done using a three-stage cluster sampling with the goal of making the sample truly representative of the entire student population [7,8].

The practice of vaporizing nicotine-infused liquid using a lithium battery was first developed by Chinese pharmacist Hon Lik in 2003 and patented internationally in the year 2007 [9,10]. Since its introduction, use and commercialization of e-cigarettes have expanded rapidly. As per a report published by the CDC on 24 October 2024, the popularity of vaping was highlighted in several ways. Between February 2020 and June 2024, the market share of disposable e-cigarettes more than doubled—from 26.0% to 58.1% of total sales [11]. In June 2024, 80.6% (17.0 million units) of e-cigarette sales were for flavors other than tobacco, such as menthol, mint, or other flavors [11,12]. The rise in the usage is phenomenal when we observe that e-cigarettes entered the U.S. market in 2006 [13,14]. In the United States, a variety of electronic nicotine delivery systems (ENDSs) are sold in about 8500 vape shops [15]. An overarching goal of current research is to probe why a youngster is vaping. In the early years (pre-2021), queries in the questionnaire were not helpful to our investigation. Things changed starting in 2021. Two distinct lines of questioning were opened. 1. Why you started vaping to begin with, with reasons; 2. What are the reasons you are currently vaping? Reasons were expanded too. Our investigation proceeds on two prongs following these distinct lines covering the years from 2021 to 2023, unlike other studies.

Most published work did piecemeal analysis either by year, year by year, or two years at a time [4,16,17,18]. Furthermore, the main reason focused on was the availability of flavors [16]. Our study is more comprehensive, covering six years at a time and trends were examined statistically via the Cochran–Armitage trend test. In some studies, several factors were attributed to the appeal of e-cigarettes among youth, including curiosity, stress relief, flavors, and peer pressure. The availability of flavored vapes is among the top 10 reasons youth report ever trying an e-cigarette. This covered the years 2013–2015 only [16]. The tobacco industry’s advertisements and promotional activities are causal to the onset of smoking in youth and young adults [17]. Importantly, while some youth perceive e-cigarettes as less harmful than combustible cigarettes [18], emerging evidence suggests significant risks, including bronchitis or asthma, altered brain development, memory impairment, risk of cardiovascular problems, and nicotine dependence [19,20].

Given these concerns, examining trends for e-cigarette use among youth and understanding the primary motivation behind initiation and continued use is crucial. This study aims to assess e-cigarette use trends among adolescents from 2018 to 2023, with a focus on four main objectives:

(1) Analyzing vaping prevalence, including overall trends, gender-specific patterns, comparisons between middle and high school students, and differences across individual grade levels.
(2) Investigating exclusive e-cigarette use, exclusive traditional cigarette smoking, and the overlap between the two behaviors.
(3) Identifying key reasons gender-wise for adolescents for first using or continuing to use e-cigarettes.
(4) A pupil is provided with a list of reasons for vaping to begin with and also when he/she becomes a habitual user. Examine how the choice is impacted by gender. This is a complex problem because a pupil can check more than one reason for starting to vaping.

Data were sourced from the CDC’s National Youth Tobacco Survey (NYTS) questionnaire, spanning the years 2018 to 2023. Background on the CDC’s survey methodology and its historical context is available on its website.

We raised a rhetorical question—Why our youth vape?—in the title of the manuscript. This question was addressed by Hill et al. [4] with clear evocation. The authors used only one year’s data, i.e., 2022 data. We wanted to see how the answers evolved over time from 2018 to 2023, encompassing the COVID-19 years. We are able to address the question because the following items were queried in the questionnaire.

All years: 2018–2023: usage

A. Have you ever used e-cigarettes, even once or twice?

Question A was amplified into two questions B and C in the years 2021–2023.

2021–2023: Initial usage

B. What was the reason you used e-cigarettes for the first time?

2021–2023: Current usage

C. What is the reason for your current use of e-cigarettes?

Trend analysis was conducted in three phases in response to Questions A–C, and the entire exercise forms the backbone of our study.

This research falls into the genre of ‘Discovery Studies.’ We are not predicting anything. Our overwhelming emphasis is to discover how certain patterns have evolved over time.

## 2. Materials and Methods

A key methodological strength of this study lies in the estimation of national population totals alongside their corresponding standard errors. For instance, in the 2021 NYTS data, which included a sample of 20,413 students, 3665 respondents reported having used e-cigarettes at least once. Extrapolating to the national population of middle and high school students—estimated at 27,563,807 for that year—the weighted total of e-cigarette users was approximately 5,308,448, with a standard error of 476,043. Details of the statistical weighting procedures and estimation techniques are provided in the Supplementary Materials.

To assess trends in e-cigarettes and their use over time, the Cochran–Armitage test for trends was employed. This non-parametric test was used to evaluate the null hypothesis of no significant change in usage rates across years versus the alternative hypothesis of a linear trend. The same approach was applied to multiple demographic and behavioral subgroup analyses.

For the trend test on the prevalence of vaping following answers to Question A, we prepared a 2 × 6 contingency table with the first row consisting of the total number of students who answered ‘yes’ to the question by year, and the second row consisting of the total number of students who did not answer ‘yes’ by year. Table S1 in the supplement was used to create the contingency table. This 2 × 6 contingency table was the basis for the Cochran–Armitage trend test. Similar tables were constructed and used for other types of trends. Tables S2–S5 are the basis of the test [21].

Next, we focus on determining trends in exclusive e-cigarette users and exclusive cigarette users over time. Again, the Cochran–Armitage trend test was applied to check on the null hypothesis of a lack of a trend. The methodology followed the same rubric as adopted for the general trend.

Now, the focus is on reasons for vaping. A table (Table 1) of frequencies was created with respect to reasons by gender, year, first use, and current use. A pupil could check more than one reason for vaping. It is not easy to interpret Table 1. The top three reasons were identified for each sex, year, first use, and current use. It turns out the same reasons showed up under each configuration.

The top three reasons in current use were as follows:

I. Peer pressure;

II. Anxiety;

III. Get high.

We created an artificial reason IV, as follows:

IV. Other reasons excluding I, II, and III.

Even in this reduced case, a pupil could check more than one reason (I, II, III, and IV). We created the following exclusive groups of reasons for further analysis:

1. I exclusively;
2. II exclusively;
3. III exclusively;
4. IV exclusively;
5. I and II exclusively;
6. I and III exclusively;
7. I and IV exclusively;
8. II and III exclusively;
9. II and IV exclusively;
10. III and IV exclusively;
11. I, II, and III exclusively;
12. I, II, and IV exclusively;
13. I, III, and IV exclusively;
14. I, II, and IV exclusively;
15. I, II, III, and IV.

Tables (Tables S6–S9) of frequencies were created by sex, year, first use, and current use. Venn diagramswere generated as the graphical manifestation of frequencies and percentages from Tables S6–S9.

Our next focus is how sex impacts the choice of reason. In a fixed year, for each choice (1–15) of the exclusive reason for current use, let

$X_{ij}=$ number of pupils who check the reasons exclusively in Group *i*, *i* = 1, 2,…, 15 and *j* = male or female, and

$$P_{ij}=\frac{X_{ij}}{\sum_{i=1}^{15}X_{ij}}.$$

It is well-known [22,23] that $U_{ij}=\sin^{-1}\left(\sqrt{P_{ij}}\right)$ is approximately normally distributed. The ANOVA method was implemented in $U_{ij}$’s to detect differences between the sexes and groups of reasons.

All computational work in the paper was carried out using the R software Version 4.3.2 (R Foundation for Statistical Computing, Vienna, Austria) and SAS Version 9.4 (SAS Institute Inc., Cary, NC, USA).

## 3. Results

### 3.1. Trend Analysis

#### 3.1.1. Overall Trend

The six-year dataset from NYTS, spanning 2018 to 2023, served as the primary source for examining the overall trend in e-cigarette use among middle and high school students in response to the survey question “Have you ever used e-cigarettes, even once or twice?” The total number of students classified as e-cigarette users is the key statistic for trend analysis.

**Figure 1:**
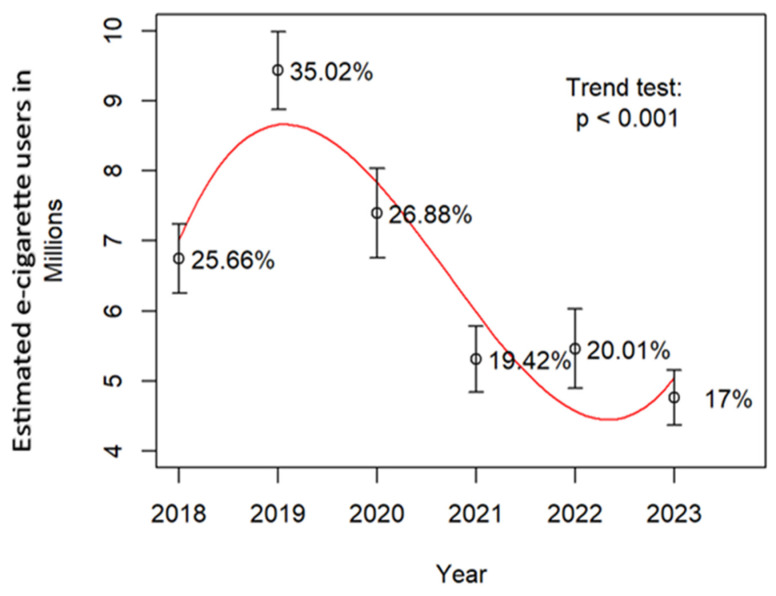
Trend in the total number of e-cigarette users with percentages, error bars, and a polynomial model.

Population estimates derived from the survey, along with associated standard errors, were analyzed and visualized using a third-degree polynomial smoothing function to illustrate the trajectory of usage over time (Figure 1). To evaluate whether the proportion of e-cigarette users significantly changed over time, the Cochran–Armitage test for trends was applied. The test yielded a *p*-value of less than 0.001, indicating a statistically significant trend. Detailed estimates and standard errors are provided in Supplementary Table S1.

#### 3.1.2. Trend by Gender

Population estimates of e-cigarette users stratified by gender are illustrated in Supplementary Figure S1, with corresponding totals and standard errors detailed in Table S2. The Cochran–Armitage trend test was applied to assess whether the population proportions of e-cigarette users differed by gender across the study years. This test yielded a *p*-value below 0.001, as shown in Figure S1, indicating a significant difference. The data revealed two distinct temporal phases: one spanning 2018 to 2020 and the other from 2021 to 2023. Separate trend analyses were conducted for each phase to capture these differing patterns.

#### 3.1.3. Trend by School Type

A comparative analysis of e-cigarette use between middle and high school students was conducted. Population estimates by school type are presented in Supplementary Figure S2, with totals and associated standard errors summarized in Table S3. These estimates were analyzed and visualized using a third-degree polynomial smoothing function to illustrate the trajectory of usage over time by school type. Figure S2a displays the trend in the total number of e-cigarette users among middle and high school students using a quadratic model, while Figure S2b presents the same trend modeled with a cubic function. The Cochran–Armitage trend test was used to assess the equality of e-cigarette usage proportions across years by school type, yielding a *p*-value less than 0.001, as reported in Figure S2, indicating statistically significant differences in trends over time.

#### 3.1.4. Trend by Grade

E-cigarette use was further analyzed by individual grade level, with middle school comprising grades 6–8 and high school including grades 9–12. Estimated population totals by grade are displayed in Supplementary Figure S3, and the corresponding values with standard errors are available in Table S4.

### 3.2. Comparison of Trend in Exclusive Cigarette and E-Cigarette Use

The survey included the question “Have you ever used a cigarette, even once or twice?” Respondents answering “Yes” were classified as cigarette users. Based on cigarette and e-cigarette usage, participants were categorized into four groups:

- Group α: Neither cigarette nor e-cigarette users;
- Group β: Cigarette users only;
- Group γ: E-cigarette users only;
- Group δ: Users of both cigarettes and e-cigarettes.

**Figure 2:**
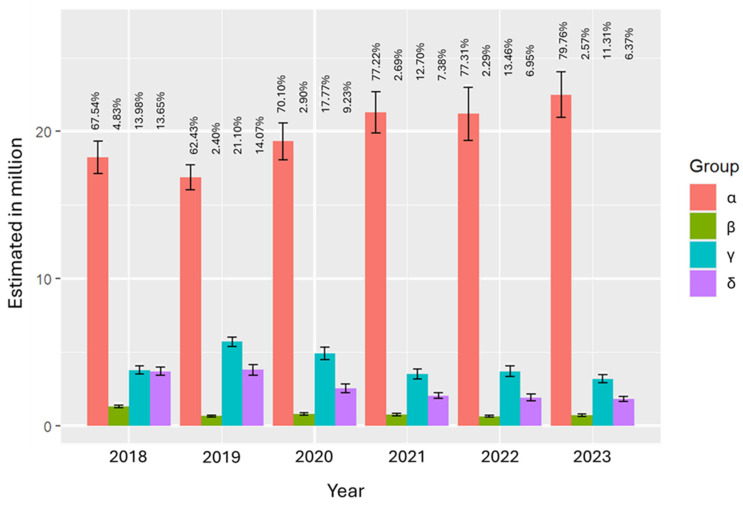
Bar plot of population estimates of total number of e-cigarette users with error bars by group.

This categorization was used to investigate claims by e-cigarette manufacturers who promote these devices as harm reduction tools for tobacco control [4,21,22,23,24]. Population totals by group and year are graphed in Figure 2, with a particular focus on the trends observed in Group γ relative to Group β. Detailed totals and standard errors are provided in Table S5.

### 3.3. Reasons for Vaping

The NYTS questionnaire included three key questions related to e-cigarette use, Questions A, B, and C, enunciated in Section 3.2. In this section, the focus was on Questions B and C.

- Question B: “What was the reason you used e-cigarettes for the first time?”
- Question C: “What is the reason for your current use of e-cigarettes?”

Statistical analyses were performed on responses to these questions, which offered respondents a checklist of 14 possible reasons for vaping.

They could check more than one reason from the set (S) of 14 reasons. For everyone in the sample, we know how many reasons the pupils checked and what they were. Let A be any subset of S. Let NA be the estimated total number of pupils who checked all the reasons in A for vaping. The total number of possible non-empty subsets A of S was 2^14^ − 1 = 16,383. The sum of all NA’s overall subset A of S was the total number of students, which was 27,563,807, for example, for the year 2021. It was impossible to handle all these numbers. We proceeded in two different ways. First, let Mi be the estimated total number of pupils who checked Reason i as a reason for vaping, i = 1, 2,…, 14. The sum of all Mi’s over i would obviously exceed 27,563,807. The number of Mi’s were easy to compile and tabulate. One disadvantage was that the numbers overlapped. For example, subjects who stated Reason 1 for vaping and subjects who gave Reason 2 for vaping overlapped. We tabulated the number of Mi’s in Table 1 by gender and year. The top three reasons (numbers) are identified by (1), (2), and (3) in the table.

The main features of Table 1 are summarized in Table 2.

‘Peer pressure’ remained as one of the top reasons for males and females when transitioning from initial use to current use, as was ‘Get high’ for males. However, ‘Curiosity’ gave way to ‘Anxiety.’ For females, ‘Anxiety’ remained as one of the top reasons in both phases of initial use and current use. However, ‘Curiosity’ gave way to ‘Get high.’

In response to Question C (reason for current e-cigarette use), different rankings of motivations emerged. Among the females, the top three reasons in 2021 and 2022 were (1) ‘Anxiety’, (2) ‘Get high’, and (3) ‘Peer Pressure’. In 2023, while ‘Anxiety’ remained the leading reason, the second and third positions were reversed, with ‘Peer Pressure’ overtaking ‘Get high’. Interestingly, ‘Get high’ remained the top reason for male students across all years. The other two reasons, ‘Anxiety’ and ‘Peer pressure’ made the cut with no one maintaining a consistent ranking. These features are extracted from Table 1 and summarized in Table 2.

Overlap is an issue in Table 1. Creating 2^14^ − 1 = 16,383 non-overlapping subsets of current users by reason is unwieldy. As a compromise, we created four categories.

Females: Initial usage: 2021–2023, by year,

I: Students who professed ‘Peer pressure’ for vaping.

II: Students who professed ‘Curiosity’ for vaping.

III: Students who professed ‘Anxiety’ for vaping.

IV: Students who professed ‘Other’ (other than ‘Peer pressure’, ‘Curiosity’, and ‘Anxiety’) for vaping.

**Figure 3:**
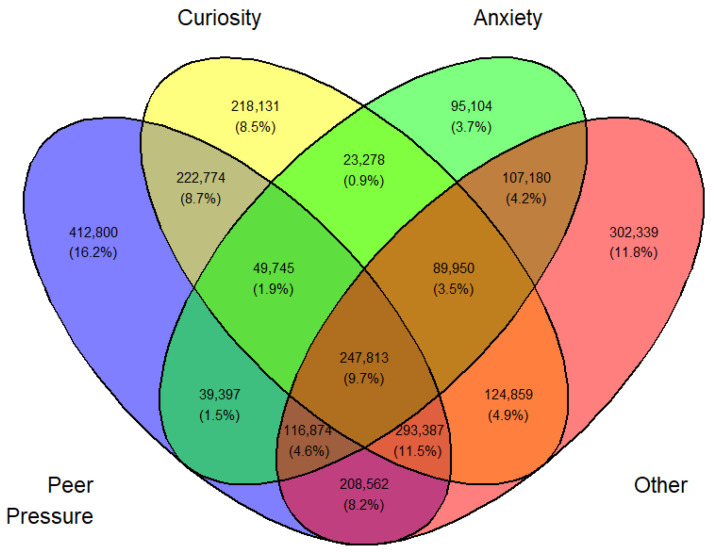
Venn diagram of first use reasons for an estimated number of female e-cigarette users in 2021. Legend: blue—peer pressure; yellow—curiosity; green—anxiety; pink—other.

Overlap was expected between sets I, II, III, and IV. They can be made disjointed (not overlapping). For example, I∩II∩III∩IV demoted the set of students who professed all four reasons for vaping. Another example, I∩II^C^∩III∩IV is the set of all students who professed ‘Peer pressure’, ‘Anxiety’, and ‘Other than the three main reasons’ but not ‘Curiosity’. The number of exclusive sets of reasons would be 2^4^ − 1 = 15. These 15 exclusive sets of reasons with corresponding frequencies and percentages are tabulated in Table S6. A Venn diagram was created for these exclusive numbers and percentages, as shown in Figure 3. As an illustration of the Venn diagram, Figure 3, the total number of students who gave the response ‘Peer pressure’ exclusively for vaping was 412,800 (16.2%), which was the largest.

In a similar fashion, a Venn diagram was created for each gender, year, and first or current use (12 in all) identified by Figure 3, Figure 4, Figure 5, Figure 6, Figure 7, Figure 8, Figure 9, Figure 10, Figure 11, Figure 12, Figure 13 and Figure 14. The legend for the corresponding reasons (color coded) is provided in the description of each Venn diagram.

In summary, the key findings are:

i. The overall number of student e-cigarette users peaked in 2019, followed by a consistent decline.
ii. From 2018 to 2021, male students exhibited higher rates of vaping than females; however, this trend reversed in 2022 and 2023.
iii. High school students reported higher rates of e-cigarette use compared to their middle school counterparts.
iv. Among all grade levels, 12th-grade students consistently had the highest rates of use.
v. The proportion of students who vape but do not smoke traditional cigarettes remains notably high, undermining claims that e-cigarettes function as cessation tools [4,24,25,26,27].
vi. Importantly, mental stress, particularly anxiety—emerged as one of the top three self-reported reasons for initiating e-cigarette use, indicating that stress management, rather than smoking cessation, may be a primary motivator.

Our findings on statistical differences between sexes and categories are presented in Table 3.

## 4. Discussion

Companies like R.J. Reynolds Tobacco Company (Winston-Salem, NC, USA) initially targeted traditional cigarette smokers [24]. Their product, Vuse, manufactured under the banner of R.J. Reynolds Vapor Company (Winston-Salem, NC, USA), is one of the most popular brands of e-cigarettes among U.S. adolescents. In October 2021, Vuse Solo became the first e-cigarette brand to receive marketing granted orders (MGOs) from the U.S. Food and Drug Administration (FDA), authorizing its marketing of their tobacco-flavored pods and was promoted as a tobacco harm reduction strategy [24,28]. However, data from the National Youth Tobacco Survey (NYTS) reveals a different story. The proportion of students who exclusively use e-cigarettes (Group γ) is roughly five times greater than those who exclusively smoke cigarettes (Group β) across all surveyed years, as detailed in Table S10. This suggests that Group γ, exclusive e-cigarette users, form the main consumer base sustaining the e-cigarette industry. Although this group is relatively small in absolute numbers, its size is much larger (about five times) than that of exclusive cigarette smokers, who might theoretically benefit from switching to e-cigarettes (Table S10).

Electronic cigarettes were first introduced to the U.S. market in 2006 [13,14,29], with the advent of “pod-mod” devices like JUUL (San Francisco, CA, USA) emerging in 2015 [29,30]. By June 2024, there were approximately 6300 e-cigarette products available in the U.S., with sales reaching USD 488.9 million that month alone [12]. Manufacturers and sellers of e-cigarettes aggressively target young people. There are few federal restrictions on e-cigarette marketing, allowing companies to promote their products through traditional outlets—such as TV and radio—despite a ban in 1971 on cigarette advertising in both outlets to reduce cigarette marketing to children. E-cigarette companies find different marketing outlets, including the internet, newspapers/magazines, TV/movies, and retail stores. In 2021, more than 75% of middle and high school students reported exposure to marketing or advertising of any nicotine or tobacco product—including e-cigarettes [27,30]. Moreover, around 2000 new vaping shops have established permanence in the market. Using the data, population estimates with standard errors are presented (Figure 1 and Table S1), showing an initial rise in usage followed by a decline. The overall trend is highly significant (*p* < 0.001). Numerous studies have documented the negative health consequences of vaping. More than 40 countries that have some type of ban on vaping—either on possession and use, sales, importation, or a combination [31]. California has enacted a ban on e-cigarettes effectively in 2026, imposing fines up to USD 500 for first offenses [32]. Similarly, The States Assembly of Jersey has unanimously approved a ban on disposable vapes, set to take effect in 2025 [33]. In Europe, as of 2013, 17 EU countries have comprehensive smoke-free laws; among them, Ireland, Greece, Bulgaria, Malta, Spain, and Hungary enforce the strictest bans on smoking in enclosed public spaces, workplaces, and public transport, with limited exceptions [34].

Gender trends in vaping reveal two phases: in the first three years, more boys vaped than girls, while in the latter three years, the trend reversed. The Cochran–Armitage trend test applied over six years confirmed significant trends (*p* < 0.001), consistent across the first three years.

When considering cigarette and e-cigarette use, four categories emerged: non-users (α), cigarette-only smokers (β), e-cigarette-only users (γ), and dual users (δ).

Examining motivations for vaping among first-time users, the top three reasons consistently were ‘Peer pressure’, ‘Curiosity’, and ‘Anxiety’. For males, ‘Anxiety’ was replaced by ‘Get high’, while ‘Peer pressure’ and ‘Curiosity’ remained important. Among current users, the primary reasons shifted slightly, with ‘Anxiety’, ‘Peer pressure’, and ‘Get high’ predominating, and ‘Curiosity’ becoming less prominent.

These findings suggest the need for gender-specific strategies to address vaping. Since ‘Anxiety’ plays a particularly significant role, especially for females, targeted interventions such as stress-reduction workshops during school hours may be beneficial. Addressing ‘Peer pressure’ remains challenging; however, providing engaging after-school activities could reduce boredom, which often drives youth toward risky behaviors in search of excitement.

Popularity of e-cigarettes is attributable to the perception that the level of toxicants in e-cigarettes is lower than that of combustible cigarettes. This is true and corroborated in Refs. [35,36,37]. Once a subject is introduced to nicotine, however small the amount is, addiction to nicotine is inescapable. Health researchers fought against cigarette manufacturers over 50 years for them to admit that smoking is harmful [38,39,40]. A similar battle is brewing against e-cigarettes. The battle will be harder because e-cigarette manufacturers target our youth to get them vaping. Our research on trends in vaping highlights the underlying magnitudes.

## 5. Conclusions

The results from NYTS showed that e-cigarettes were usually the first tobacco product used by adolescents, suggesting that vaping may act as a gateway to further drug use. All forms of smoking should be prohibited for individuals under the age of 21, in accordance with national laws [41,42]. New Jersey law states that a person who sells or offers a tobacco product to a person under 21 years of age shall pay a penalty of up to USD 1000 and may be subject to a license suspension or revocation [43]. Despite this, many young students continue to vape. Smoking, in one form or another, remains a persistent issue. Education about the potential harm of vaping, especially when initiated at a young and impressionable age, is a critical tool in the fight against youth vaping. Although the data showed a decline in vaping rates, more time is needed to confirm whether this downward trend is sustained. This study has focused on understanding the reasons behind vaping, which can inform the development of targeted educational interventions. Since anxiety and stress are among the leading reasons youth begin vaping, a thorough exploration of the root causes of these issues is essential.

There were three goals spelled out: (1) Time trend of several types parsing answers to Question A. A downward overall trend was perceived. (2) Time trend of exclusive e-cigarette users and of cigarette users. Exclusive e-cigarette users outpaced substantially exclusive cigarette users. (3) Parsing reasons for vaping. Distinct patterns emerge between first time users and current users.

## 6. Limitations

The questionnaire was self-administered. There was no way to verify the answers to questions. We assumed that pupils answered the questions truthfully. Biases and confounders could creep into analyses if this assumption is violated. To address biases, one recourse is bootstrapping data in all inferences. We have not pursued bootstraps. However, the veracity of reasons for vaping is hard to question. There is no reason to suspect the answers. The top reasons were expected. Our analysis provided numbers and percentages behind the reasons.

## Figures and Tables

**Figure 4:**
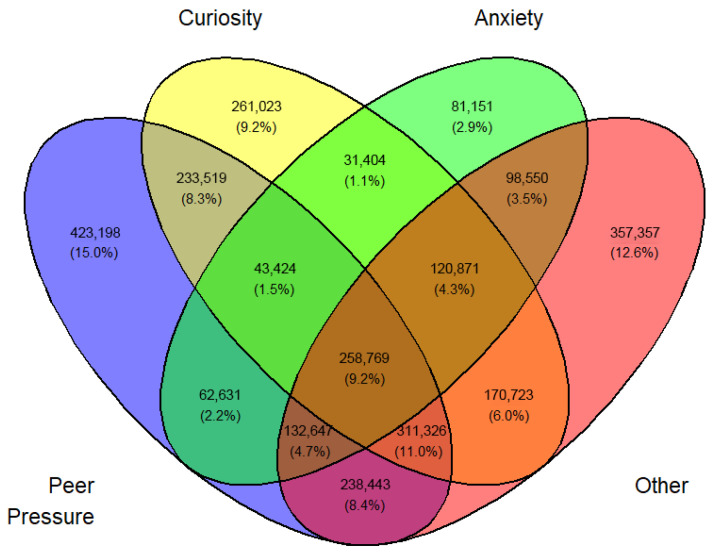
Venn diagram of first use reasons for an estimated number of female e-cigarette users in 2022. Legend: blue—peer pressure; yellow—curiosity; green—anxiety; pink—other.

**Figure 5:**
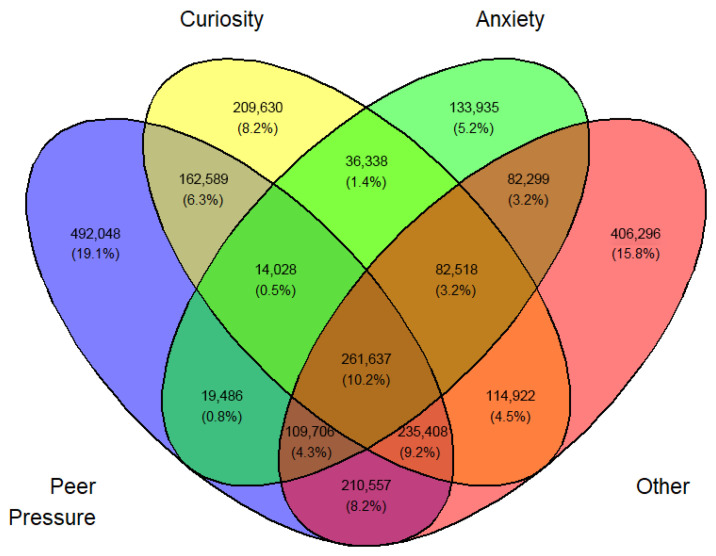
Venn diagram of first use reasons for an estimated number of female e-cigarette users in 2023. Legend: blue—peer pressure; yellow—curiosity; green—anxiety; Pink—other.

**Figure 6:**
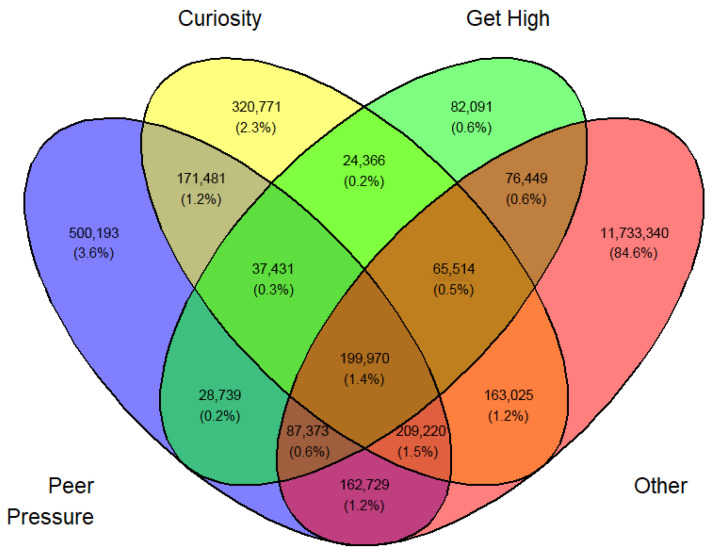
Venn diagram of first use reasons for an estimated number of male e-cigarette users in 2021. Legend: blue—peer pressure; yellow—curiosity; green—get high; pink—other.

**Figure 7:**
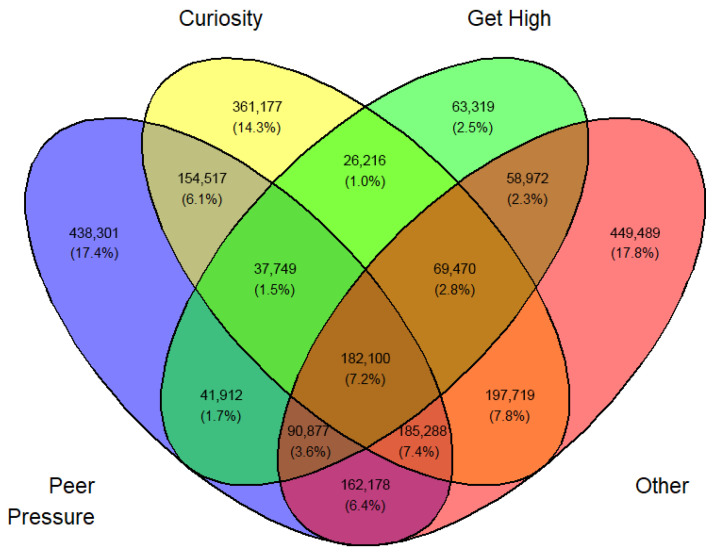
Venn diagram of first use reasons for an estimated number of male e-cigarette users in 2022. Legend: blue—peer pressure; yellow—curiosity; green—get high; pink—other.

**Figure 8:**
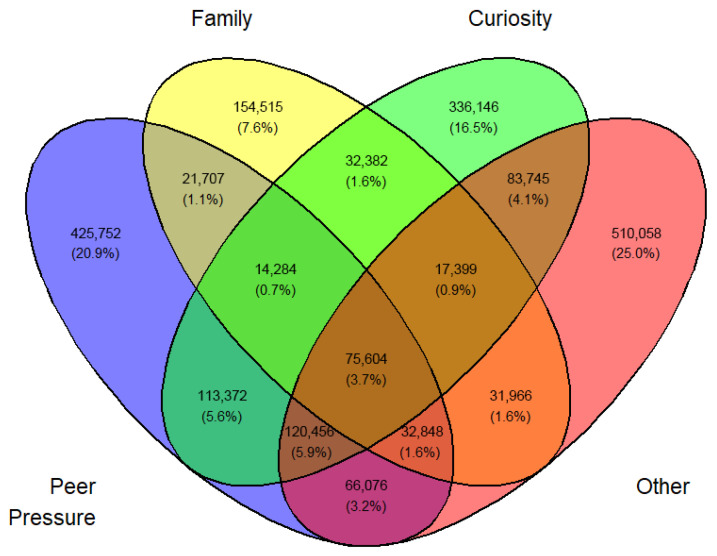
Venn diagram of first use reasons for an estimated number of male e-cigarette users in 2023. Legend: blue—peer pressure; yellow—family used; green—curiosity; pink—other.

**Figure 9:**
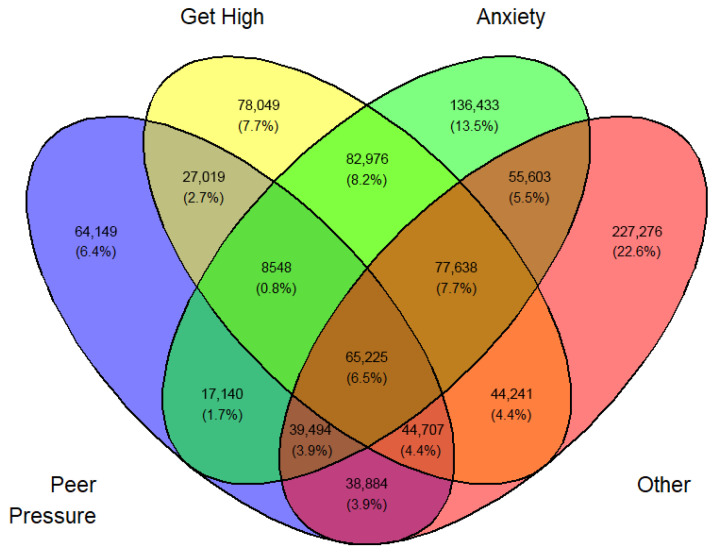
Venn diagram of current use reasons for an estimated number of female e-cigarette users in 2021. Legend: blue—peer pressure; yellow—get high; green—anxiety; pink—other.

**Figure 10:**
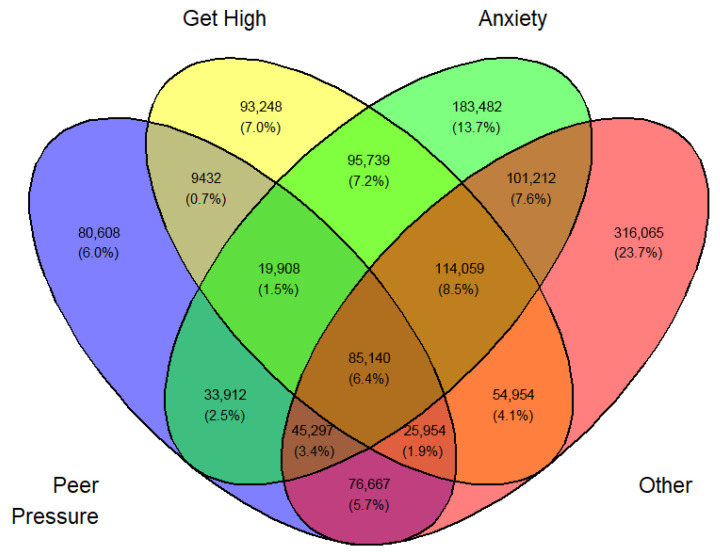
Venn diagram of current use reasons for an estimated number of female e-cigarette users in 2022. Legend: blue—peer pressure; yellow—get high; green—anxiety; pink—other.

**Figure 11:**
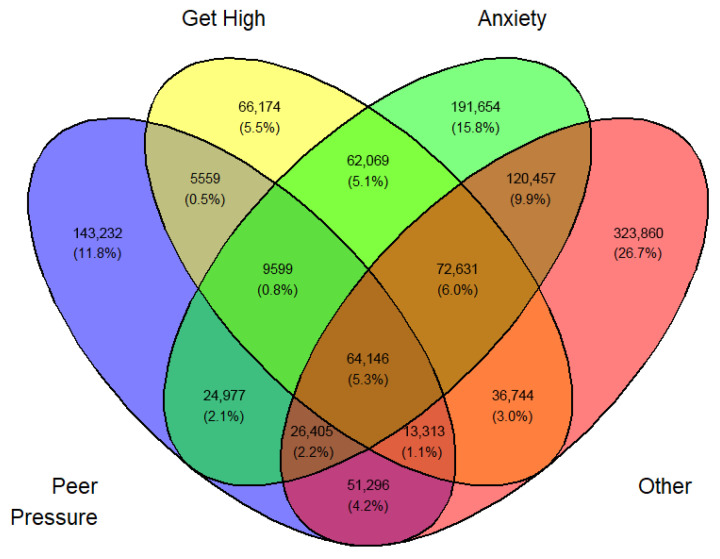
Venn diagram of current use reasons for an estimated number of female e-cigarette users in 2023. Legend: blue—peer pressure; yellow—get high; green—anxiety; pink—other.

**Figure 12:**
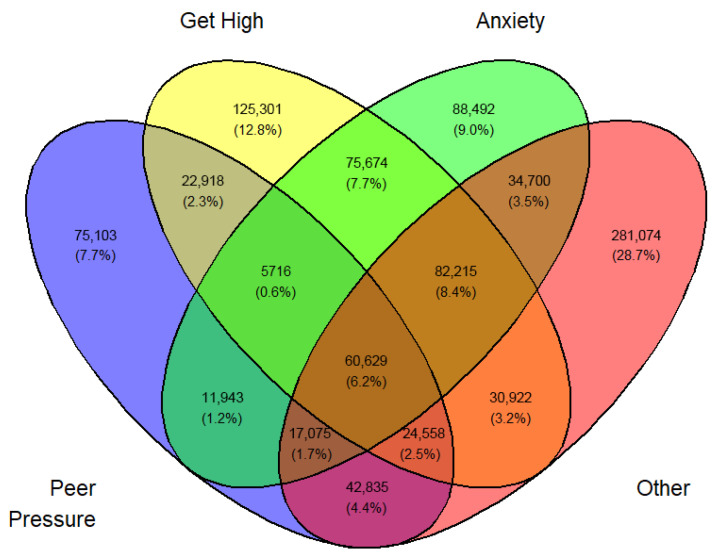
Venn diagram of current use reasons for an estimated number of male e-cigarette users in 2021. Legend: blue—peer pressure; yellow—get high; green—anxiety; pink—other.

**Figure 13:**
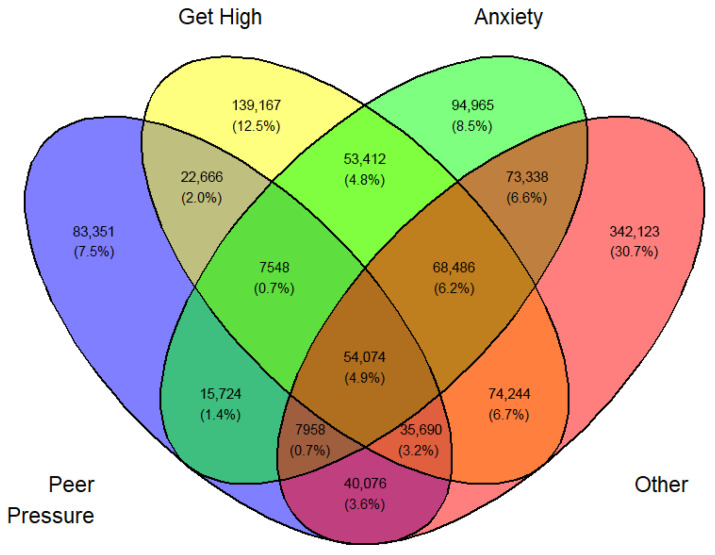
Venn diagram of current use reasons for an estimated number of male e-cigarette users in 2022. Legend: blue—peer pressure; yellow—get high; green—anxiety; pink—other.

**Figure 14:**
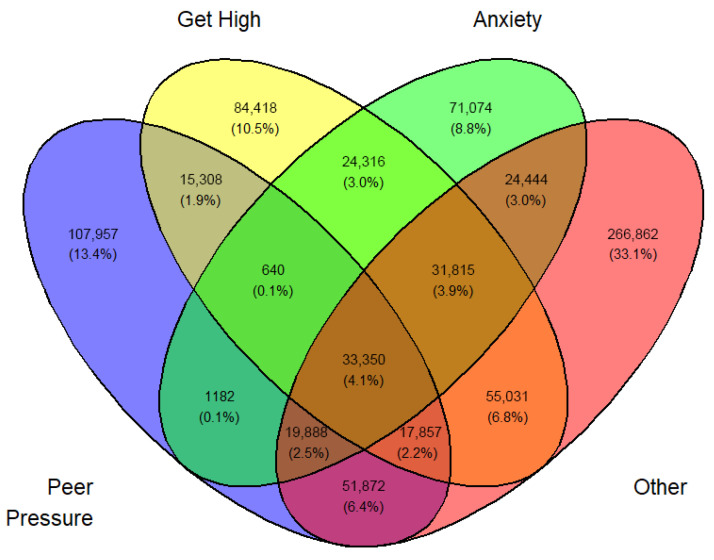
Venn diagram of current use reasons for an estimated number of male e-cigarette users in 2023. Legend: blue—peer pressure; yellow—get high; green—anxiety; pink—other.

**Table 1:** Estimated total number of pupils under each reason for vaping by gender and year differentiated by first use and current use; top three reasons are identified in parentheses as (1), (2), and (3).

Question	Reason for First Use						Reason for Current Use					
Year	2021		2022		2023		2021		2022		2023	
Reason	Male	Female	Male	Female	Male	Female	Male	Female	Male	Female	Male	Female
**Peer pressure**	1,397,140 (1)	1,591,356 (1)	1,292,926 (1)	1,703,960 (1)	870,112 (1)	1,505,463 (1)	267,091 (3)	376,923 (3)	267,091 (3)	376,923 (3)	248,057 (2)	338,531 (2)
**Family used**	414,410	543,345	479,423	698,610	380,707 (3)	679,469	89,345	121,752	89,345	121,752	61,778	129,352
**Quit smoking**	71,919	59,593	70,024	40,197	74,515	42,804	41,456	32,377	41,456	32,377	55,222	38,512
**Costs less**	80,827	28,630	84,746	36,697	66,440	39,437	49,406	53,702	49,406	53,702	63,012	53,033
**Easier to get**	132,816	113,215	157,161	124,494	114,816	145,505	60,090	86,845	60,090	86,845	77,024	135,593
**Ads**	123,394	111,458	152,755	181,465	103,977	129,616	36,906	39,825	36,906	39,825	45,480	33,168
**Less harmful**	258,211	169,299	207,955	184,347	111,989	198,543	115,603	99,907	115,603	99,907	77,784	167,452
**Flavors**	310,477	385,656	337,139	456,994	191,949	367,716	140,517	194,153	140,517	194,153	123,598	195,577
**Can be masked**	235,357	321,884	302,860	367,231	162,583	305,983	151,638	222,130	151,638	222,130	97,039	196,474
**Do tricks**	415,138	434,866	475,772	475,619	246,441	396,980	214,816	260,605	214,816	260,605	157,083	238,812
**Curiosity**	1,191,782 (2)	1,269,940 (2)	1,214,240 (2)	1,431,062 (2)	793,400 (2)	1,117,074 (2)	118,738	194,762	118,738	194,762	130,084	124,538
**Anxiety**	523,571	769,345 (3)	495,790	829,450 (3)	281,896	739,950 (3)	375,509 (2)	678,753 (1)	375,509 (2)	678,753 (1)	206,714 (3)	571,941 (1)
**Get high**	601,938 (3)	604,630	570,619 (3)	655,439	376,725 (3)	546,496	455,291 (1)	498,438 (2)	455,291 (1)	498,438 (2)	262,740 (1)	330,238 (3)
**None above**	313,987	227,257	254,784	206,251	168,396	151,996	244,677	216,287	244,677	216,287	120,560	185,874

**Table 2:** Top three reasons for vaping in order of importances.

Question	Reason for First Use			Reason for Current Use		
Year	2021	2022	2023	2021	2022	2023
**Male**	**Peer pressure**	**Peer pressure**	**Peer pressure**	**Get high**	**Get high**	**Get high**
	**Curiosity**	**Curiosity**	**Curiosity**	**Anxiety**	**Anxiety**	**Peer pressure**
	**Get high**	**Get high**	**Family used and Get high**	**Peer pressure**	**Peer pressure**	**Anxiety**
**Female**	**Peer pressure**	**Peer pressure**	**Peer pressure**	**Anxiety**	**Anxiety**	**Anxiety**
	**Curiosity**	**Curiosity**	**Curiosity**	**Get high**	**Get high**	**Peer pressure**
	**Anxiety**	**Anxiety**	**Anxiety**	**Peer pressure**	**Peer pressure**	**Get high**

**Table 3:** ANOVA table by gender and category for years 2021–2023 (current use).

Year		Df	Sum Sq	Mean Sq	F Value	Pr (>F)	
2021	Sex	1	2.66 × 10^7^	2.66 × 10^7^	0.081	0.78	
	Category	14	1.03 × 10^11^	7.33 × 10^9^	22.363	3.59 × 10^−7^	***
	Residuals	14	4.59 × 10^9^	3.28 × 10^8^			
2022	Sex	1	1.66 × 10^9^	1.66 × 10^9^	2.761	0.119	
	Category	14	1.69 × 10^11^	1.21 × 10^10^	20.168	7.00 × 10^−7^	***
	Residuals	14	8.39 × 10^9^	6.00 × 10^8^			
2023	Sex	1	5.50 × 10^9^	5.50 × 10^9^	6.76	0.021	*
	Category	14	1.52 × 10^11^	1.09 × 10^10^	13.37	9.29 × 10^−6^	***
	Residuals	14	1.14 × 10^10^	8.13 × 10^8^			

We used the British system of recording the degree of significance. A ‘***’ designation means if the *p*-value is less than 0.001. A ‘*’ designation means if the *p*-value is in between 0.01 and 0.05. No significant differences were detected between the sex in 2021 or 2022. However, categories were significantly different in each year.

## Data Availability

The original data presented in the study are openly available on the CDC website.

## Supplementary Materials

The following supporting information can be downloaded at: https://www.mdpi.com/article/10.3390/medicina62010223/s1, Table S1. Estimates with standard errors of total number of e-cigarette users. Figure S1. Trend of total numbers of e-cigarette users by gender. Table S2. Estimates of total number of e-cigarette users with standard errors and percentages by gender. Figure S2. Trend of total numbers of e-cigarette users by school type among middle and high school students. Figure S2a. Trend of total numbers of e-cigarette users among middle and high school students with quadratic trend. Figure S2b. Trend of total numbers of e-cigarette users among middle and high school students with cubic trend. Table S3. Estimates of total number of e-cigarette users with standard errors and percentages by school type. Figure S3. Trend of total numbers of e-cigarette users by grade among middle and high school students. Table S4. Estimates of total number of e-cigarette users and percentages across the grades. Table S5. Estimates of total number of E-cigarette users and percentages by groups. Table S6. Reasons for vaping first time—Females. Table S7. Reasons for vaping first time—Males. Table S8. Reasons for vaping currently—Females. Table S9. Reasons for vaping currently—Males. Table S10. Percentage of cigarette and e-cigarette users exclusively and the ratio.

## Abbreviations

The following abbreviations are used in this manuscript:

ENDS	Electronic nicotine delivery systems
NYTS	National Youth Tobacco Survey
CDC	Centers for Disease Control and Prevention
FDA	Food and Drug Administration
