# Supplementary material for: Why Our Youth Vape?—A Trend Analysis Based on Cross-Sectional Annual Surveys of Middle and High School Students in the U.S
Source: Medicina (Kaunas). 2026 Jan 21;62(1):223. doi: 10.3390/medicina62010223 (PMC12844295; doi:10.3390/medicina62010223)
Supplement: Supplementary file 1 [file medicina-62-00223-s001.zip › medicina-3977176-supplementary.pdf]

SUPPLEMENT MATERIALS

Title: **Why Our Youth Vape?—A Trend Analysis Based on Cross-Sectional Annual Surveys of Middle and High School Students in the U.S.**

Table S1. Estimates with standard errors of total number of e-cigarette users

**Year**	**Total number of pupils**	**Estimates of total number of e-cigarette users**	**Standard error**	**Percentage of users**
**2018**	27,011,216	6,748,803	492,538	25.66%
**2019**	27,011,216	9,438,154	559,986	35.02%
**2020**	27,563,807	7,395,204	642,050	26.88%
**2021**	27,563,807	5,308,448	476,043	19.42%
**2022**	27,412,530	5,457,655	565,146	20.01%
**2023**	28,201,526	4,756,837	395,607	17.00%

Figure S1: Trend of total numbers of e-cigarette users by gender

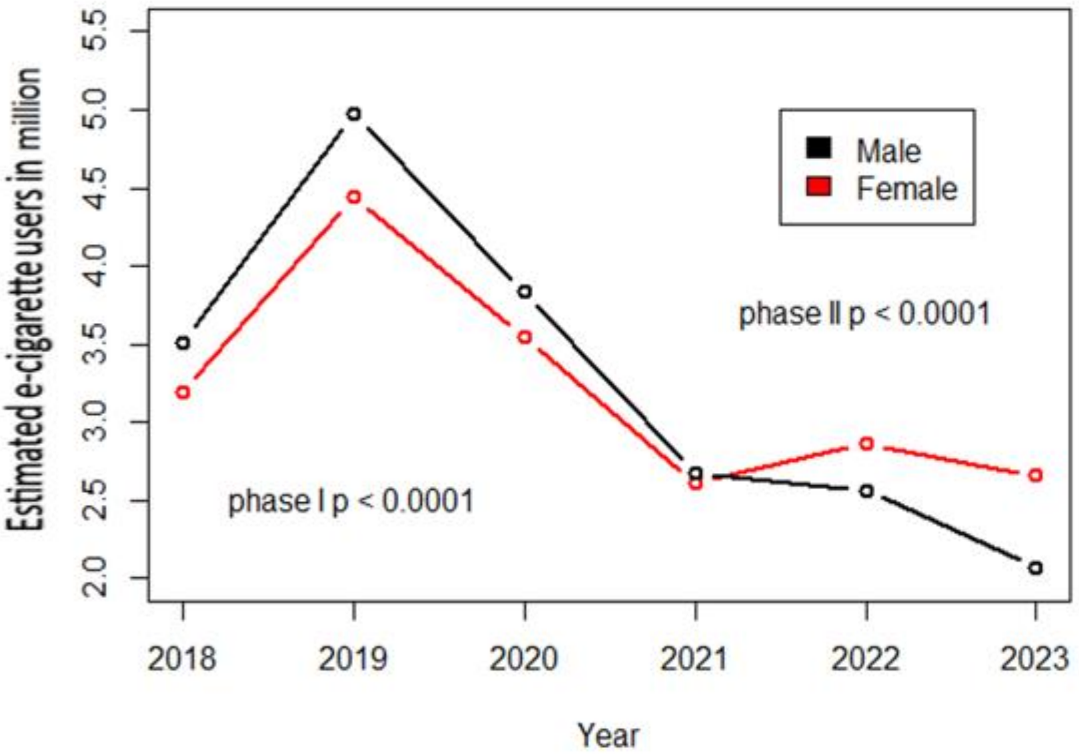

Table S2. Estimates of total number of e-cigarette users with standard errors and percentages by gender								
**Year**	**Female**	**Standard error**	**Percentages Females vs Males**	**Percentages Females vs Total**	**Male**	**Standard error**	**Percentages Males vs Females**	**Percentages Males vs Total**
**2018**	3,189,081	250,667	47.61	24.53	3,509,135	262,183	52.39	26.78
**2019**	4,437,690	254,253	47.17	34.47	4,969,703	379,369	52.83	35.66
**2020**	3,546,930	320,113	48.06	26.19	3,832,523	343,034	51.94	27.57
**2021**	2,612,319	228,832	49.41	20.09	2,675,013	278,874	50.59	18.81
**2022**	2,861,375	290,432	52.72	21.58	2,566,586	285,183	47.28	18.56
**2023**	2,658,546	229,386	56.20	19.41	2,071,877	206,157	43.80	15.67

Figure S2:Trend of total numbers of e-cigarette users by school type among middle and high school students

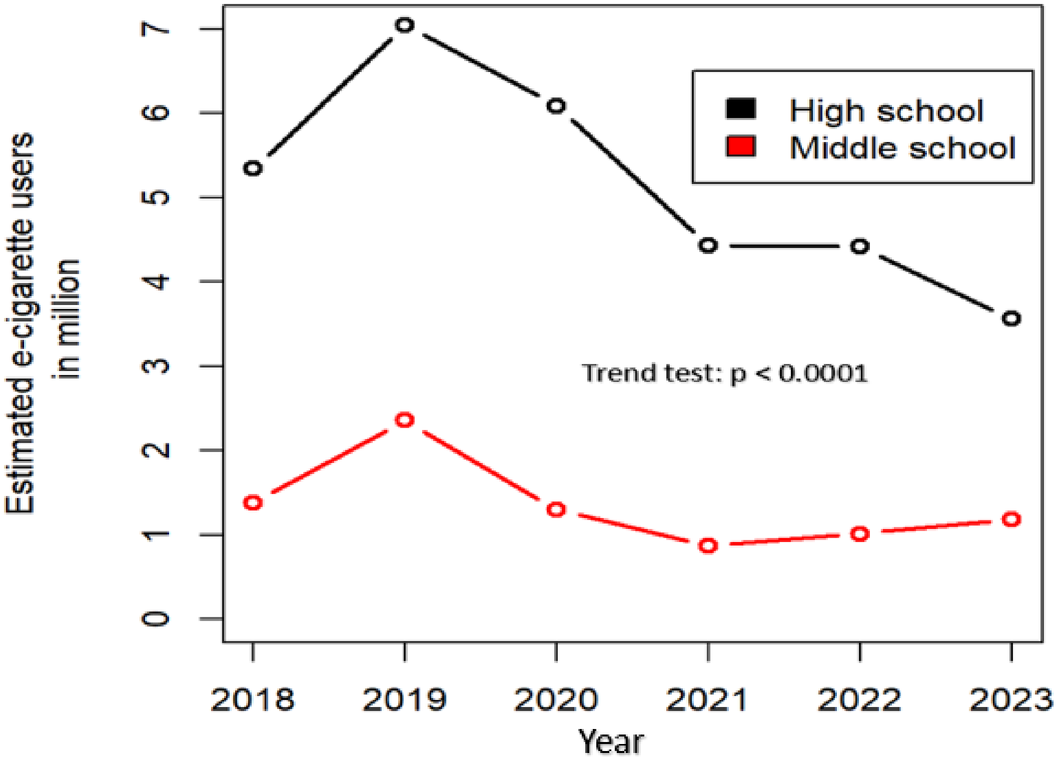

Figure S2(a):

Trend of total numbers of e-cigarette users among middle and high school students with quadratic trend

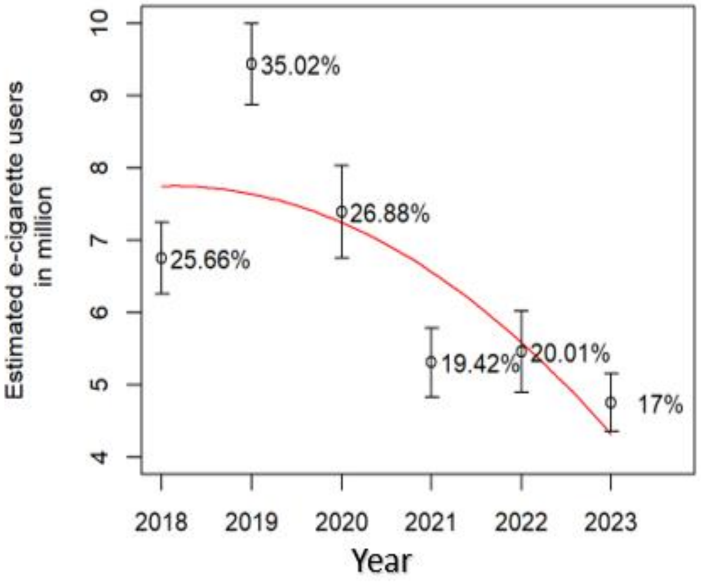

Figure S2(b):

Trend of total numbers of e-cigarette users among middle and high school students with cubic trend

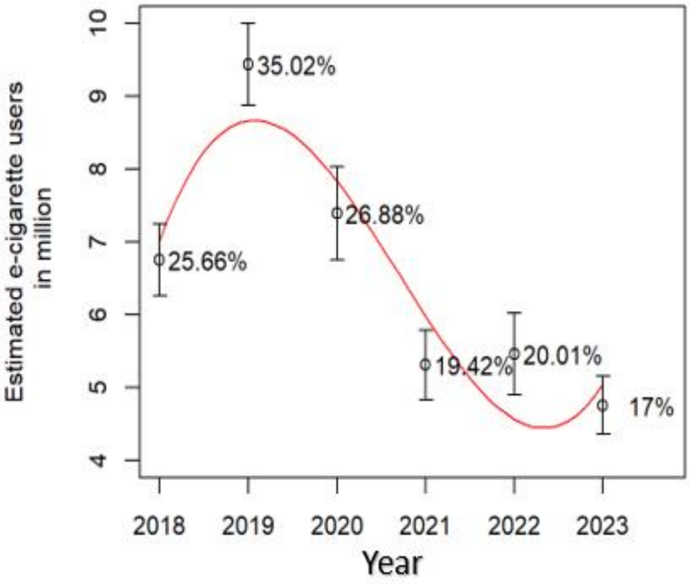

Table S3. Estimates of total number of e-cigarette users with standard errors and percentages by school type								
**Year**	**Middle School**	**Standard error**	**Percentages Middle School vs High School**	**Percentages Middle School vs Total**	**High School**	**Standard error**	**Percentages High School vs Middle School**	**Percentages High School vs Total**
**2018**	1,381,981	118,606	20.56	11.96	5,340,125	458,545	79.44	36.56
**2019**	2,356,016	176,806	25.05	19.90	7,047,815	520,614	74.95	46.95
**2020**	1,294,826	160,120	17.54	10.79	6,086,172	615,667	82.46	39.38
**2021**	866,857	101,891	16.36	7.28	4,430,306	440,424	83.64	28.86
**2022**	1,012,464	120,887	18.65	8.52	4,416,001	517,704	81.35	28.91
**2023**	1,179,268	162,658	24.89	9.70	3,557,896	398,899	75.11	22.61

Figure S3:Trend of total numbers of e-cigarette users by grade among high school students

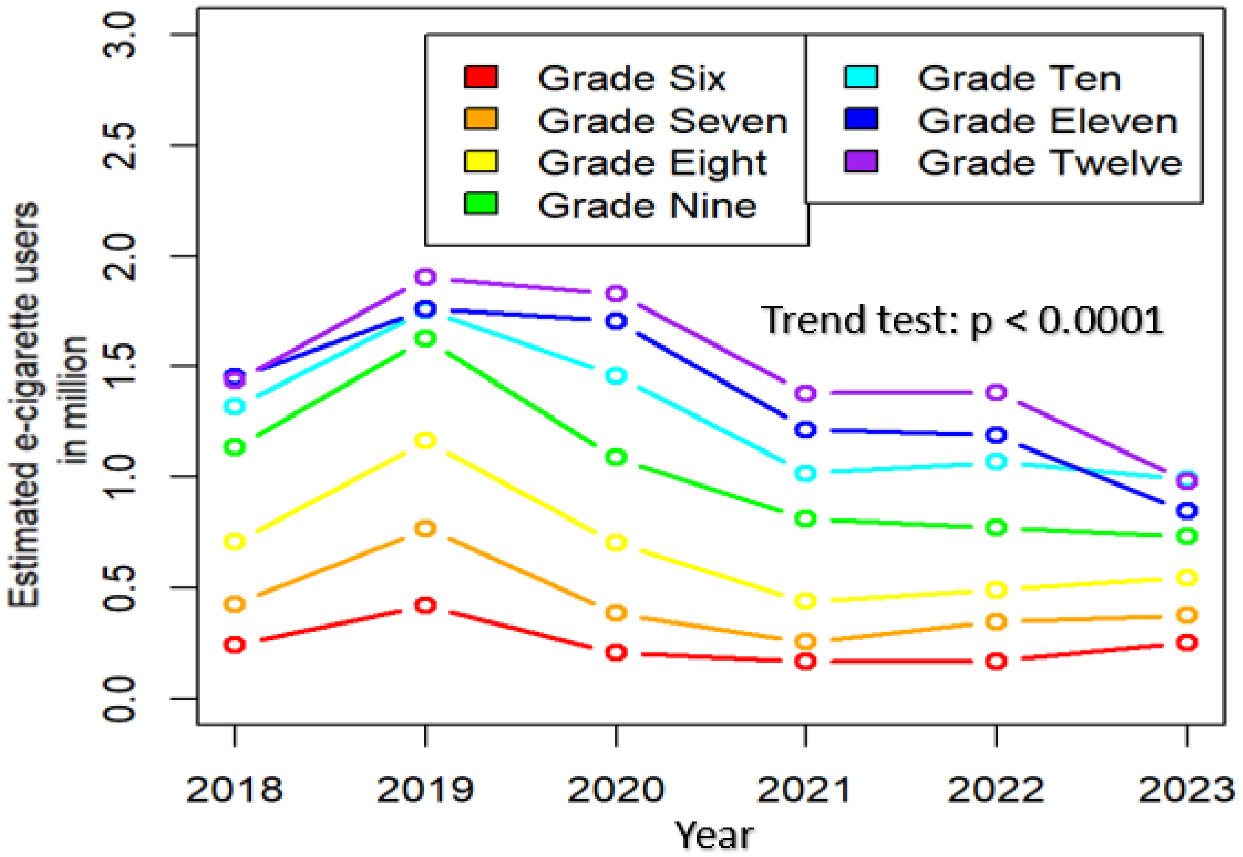

Table S4. Estimates of total number of e-cigarette users and percentages across the grades							
**Year\Grade**	**Six**	**Seven**	**Eight**	**Nine**	**Ten**	**Eleven**	**Twelve**
**2018**	245,768<br>3.66%	428,039<br>6.37%	708,174<br>10.54%	1,134,975<br>16.88%	1,317,963<br>19.61%	1,450,671<br>21.58%	1,436,517<br>21.37%
**2019**	422,156<br>4.49%	768,293<br>8.17%	1,165,567<br>12.39%	1,625,779<br>17.29%	1,757,631<br>18.69%	1,761,323<br>18.73%	1,903,081<br>20.24%
**2020**	207,104<br>2.81%	385,708<br>5.23%	702,013<br>9.51%	1,088,815<br>14.75%	1,457,876<br>19.75%	1,707,397<br>23.13%	1,832,083<br>24.82%
**2021**	169,394<br>3.20%	258,152<br>4.87%	439,310<br>8.29%	815,303<br>15.39%	1,017,773<br>19.21%	1,216,688<br>22.97%	1,380,542<br>26.06%
**2022**	170,400<br>3.14%	348,911<br>6.43%	493,153<br>9.08%	771,582<br>14.21%	1,069,323<br>19.70%	1,189,480<br>21.91%	1,385,616<br>25.53%
**2023**	253,915<br>5.36%	377,849<br>7.98%	547,504<br>11.56%	735,973<br>15.54%	993,685<br>20.98%	847,081<br>17.88%	981,158<br>20.71%

Table S5. Estimates of total number of E-cigarette users and percentages by groups					
**Year**	**Group**	**α**	**β**	**γ**	**δ**
**2018**	Estimate of total	18,244,141	1,304,910	3,776,282	3,685,882
	Standard error	1,095,383	93,356	275,524	276,468
	Percentages	67.54	4.83	13.98	13.65
**2019**	Estimate of total	16,863,923	648,093	5,698,035	3,801,166
	Standard error	848,295	68,546	306,044	357,552
	Percentages	62.43	2.40	21.10	14.07
**2020**	Estimate of total	19,321,605	800,042	4,896,712	2,545,448
	Standard error	1,248,085	93,876	419,050	297,512
	Percentages	70.10	2.90	17.77	9.23
**2021**	Estimate of total	21,285,676	742,207	3,501,033	2,034,891
	Standard error	1,409,125	70,507	330,482	179,542
	Percentages	77.22	2.69	12.70	7.38
**2022**	Estimate of total	21,191,530	627,284	3,688,790	1,904,925
	Standard error	1,797,741	59,372	370,892	227,515
	Percentages	77.31	2.29	13.46	6.95
**2023**	Estimate of total	22,493,477	723,642	3,188,551	1,795,856
	Standard error	1,553,619	88,096	274,046	164,359
	Percentages	79.76	2.57	11.31	6.37

Table S6. Reasons for vaping first time – Females						
**Peer Pressure**	**Curiosity**	**Anxiety**	**Other**	**Year 2021**	**Year 2022**	**Year 2023**
1	1	1	1	247,813<br>9.71%	258,769<br>9.16%	261,637<br>10.17%
1	1	1	0	49,745<br>1.95%	43,424<br>1.54%	14,028<br>0.55%
1	1	0	1	293,387<br>11.5%	311,326<br>11.02%	235,408<br>9.15%
1	1	0	0	222,774<br>8.73%	233,519<br>8.27%	162,589<br>6.32%
1	0	1	1	116,874<br>4.58%	132,647<br>4.70%	109,706<br>4.27%
1	0	1	0	39,397<br>1.54%	62,631<br>2.22%	19,486<br>0.76%
1	0	0	1	208,562<br>8.17%	238,443<br>8.44%	210,557<br>8.19%
1	0	0	0	412,800<br>16.17%	423,198<br>14.98%	492,048<br>19.14%
0	1	1	1	89,950<br>3.52%	120,871<br>4.28%	82,518<br>3.21%
0	1	1	0	23,278<br>0.91%	31,404<br>1.11%	36,338<br>1.41%
0	1	0	1	124,859<br>4.89%	170,723<br>6.04%	114,922<br>4.47%
0	1	0	0	218,131<br>8.55%	261,023<br>9.24%	209,630<br>8.15%
0	0	1	1	107,180<br>4.20%	98,550<br>3.49%	82,299<br>3.20%
0	0	1	0	95,104<br>3.73%	81,151<br>2.87%	133,935<br>5.21%
0	0	0	1	302,339<br>11.85%	357,357<br>12.65%	406,296<br>15.80%

Table S7. Reasons for vaping first time – Males						
**Peer Pressure**	**Curiosity**	**Get high**	**Other**	**Year 2021**	**Year 2022**	**Year 2023**
1	1	1	1	199,970<br>1.44%	182,100<br>7.23%	75,604<br>3.71%
1	1	1	0	37,431<br>0.27%	37,749<br>1.50%	14,284<br>0.70%
1	1	0	1	209,220<br>1.51%	185,288<br>7.35%	32,848<br>1.61%
1	1	0	0	171,481<br>1.24%	154,517<br>6.13%	21,707<br>1.07%
1	0	1	1	87,373<br>0.63%	90,877<br>3.61%	120,456<br>5.92%
1	0	1	0	28,739<br>0.21%	41,912<br>1.66%	113,372<br>5.57%
1	0	0	1	162,729<br>1.17%	162,178<br>6.44%	66,076<br>3.24%
1	0	0	0	500,193<br>3.61%	438,301<br>17.4%	425,752<br>20.91%
0	1	1	1	65,514<br>0.47%	69,470<br>2.76%	17,399<br>0.85%
0	1	1	0	24,366<br>0.18%	26,216<br>1.04%	32,382<br>1.59%
0	1	0	1	163,025<br>1.18%	197,719<br>7.85%	31,966<br>1.57%
0	1	0	0	320,771<br>2.31%	361,177<br>14.34%	154,515<br>7.59%
0	0	1	1	76,449<br>0.55%	58,972<br>2.34%	83,745<br>4.11%
0	0	1	0	82,091<br>0.59%	63,319<br>2.51%	336,146<br>16.51%
0	0	0	1	11,733,340<br>84.64%	449,489<br>17.84%	510,058<br>25.05%

Table S8. Reasons for vaping currently – Females						
**Peer Pressure**	**Get high**	**Anxiety**	**Other**	**Year 2021**	**Year 2022**	**Year 2023**
1	1	1	1	65,225<br>6.47%	85,140<br>6.37%	64,146<br>5.29%
1	1	1	0	8,548<br>0.85%	19,908<br>1.49%	9,599<br>0.79%
1	1	0	1	44,707<br>4.44%	25,954<br>1.94%	13,313<br>1.10%
1	1	0	0	27,019<br>2.68%	9,432<br>0.71%	5,559<br>0.46%
1	0	1	1	39,494<br>3.92%	45,297<br>3.39%	26,405<br>2.18%
1	0	1	0	17,140<br>1.70%	33,912<br>2.54%	24,977<br>2.06%
1	0	0	1	38,884<br>3.86%	76,667<br>5.74%	51,296<br>4.23%
1	0	0	0	64,149<br>6.37%	80,608<br>6.03%	143,232<br>11.82%
0	1	1	1	77,638<br>7.71%	114,059<br>8.54%	72,631<br>5.99%
0	1	1	0	82,976<br>8.24%	95,739<br>7.17%	62,069<br>5.12%
0	1	0	1	44,241<br>4.39%	54,954<br>4.11%	36,744<br>3.03%
0	1	0	0	78,049<br>7.75%	93,248<br>6.98%	66,174<br>5.46%
0	0	1	1	55,603<br>5.52%	101,212<br>7.58%	120,457<br>9.94%
0	0	1	0	136,433<br>13.54%	183,482<br>13.74%	191,654<br>15.81%
0	0	0	1	227,276<br>22.56%	316,065<br>23.66%	323,860<br>26.72%

Table S9. Reasons for vaping currently – Males						
**Peer Pressure**	**Get high**	**Anxiety**	**other**	**Year 2021**	**Year 2022**	**Year 2023**
1	1	1	1	60,629<br>6.19%	54,074<br>4.86%	33,350<br>4.14%
1	1	1	0	5,716<br>0.58%	7,548<br>0.68%	640<br>0.08%
1	1	0	1	24,558<br>2.51%	35,690<br>3.21%	17,857<br>2.22%
1	1	0	0	22,918<br>2.34%	22,666<br>2.04%	15,308<br>1.90%
1	0	1	1	17,075<br>1.74%	7,958<br>0.72%	19,888<br>2.47%
1	0	1	0	11,943<br>1.22%	15,724<br>1.41%	1,182<br>0.15%
1	0	0	1	42,835<br>4.37%	40,076<br>3.60%	51,872<br>6.44%
1	0	0	0	75,103<br>7.67%	83,351<br>7.49%	107,957<br>13.39%
0	1	1	1	82,215<br>8.40%	68,486<br>6.15%	31,815<br>3.95%
0	1	1	0	75,674<br>7.73%	53,412<br>4.80%	24,316<br>3.02%
0	1	0	1	30,922<br>3.16%	74,244<br>6.67%	55,031<br>6.83%
0	1	0	0	125,301<br>12.80%	139,167<br>12.51%	84,418<br>10.47%
0	0	1	1	34,700<br>3.54%	73,338<br>6.59%	24,444<br>3.03%
0	0	1	0	88,492<br>9.04%	94,965<br>8.53%	71,074<br>8.82%
0	0	0	1	281,074<br>28.71%	342,123<br>30.74%	266,862<br>33.11%

**Table S10. Percentage of cigarette and e-cigarette users exclusively and the ratio**			
**Year**	**Group β<br><br>Exclusive<br><br>Cigarette users**	**Group γ<br><br>Exclusive<br><br>E-Cigarette users**	**Ratio (γ/β)**
**2018**	4.83	13.98	2.89
**2019**	2.40	21.10	8.79
**2020**	2.90	17.77	6.13
**2021**	2.69	12.70	4.72
**2022**	2.29	13.46	5.88
**2023**	2.57	11.31	4.40
**Average**	2.95	15.05	5.47
